# Personal

**Published:** 1916-08

**Authors:** 


### PERSONAL.

---

**Announcement of removal, travel, and other matters of interest are requested. Please report errors in the listing of any physician in the State and other directories, that we may co-operate with the State Society in securing a correct list.**

---

Dr. G. B. Broad and family, of Syracuse, are spending the month of July at Star Lake.

---

Dr. Fred. Flaherty and family, of Syracuse, are spending the month of July in the Adirondacks.

---

Dr. I. H. Levy, of Syracuse, was elected President of Lake Kueka Medical Association.

---

Dr. Geo. K. Smith has just finished his service as interne at St. Joseph's Hospital, Syracuse, N. Y., and is now associated with his father, Dr. F. W. Smith, 1350 So. Salina Street, Syracuse, N. Y.

---

Dr. Roland O. Meisenbach of Buffalo left July 15 for a motor tour to Maine.

---

Dr. Julius Ullman of Buffalo spent the latter half of July in the Adirondacks.

---

Dr. A. C. Way of Perry is at Saranac Lake.

---

Dr. George R. Critchlow and Dr. R. Montfort Schley of Buffalo, attended the training camp at Plattsburg during July.

---

Dr. Clayton W. Greene, of Buffalo, 1st Lt. U. S. Reserve Medical Corps, was ordered to Columbus Barracks, O., in July, to administer typhoid vaccine.

---

Dr. H. H. Glosser, Buffalo, 1st Lt.; G. McKay Hall of Buffalo 1st Lt. and Arthur C. Scaefer, Capt., are on duty with the 74th Regiment on the border.

---

Dr. Walter C. Montgomery of N. Y. City has been appointed Surgeon and Major in the 74th Reg. vice Dr. Edwin L. Beebe of Buffalo, Major, transferred to the 12th Reg.

---

Dr. Henry Adsit of Buffalo, Capt. 1st Cavalry, attached to Troop I is with that body on the border.

Dr. John D. Howland, Lt. Col. of the 65th Reg., now the 3d Artillery, is with the Regiment at Camp Whitman.

---

Dr. N. Victoria Chappell and Miss E. Mae Chappell of Buffalo returned from a western trip about the first of July.

---

Dr. C. W. Hennington of Rochester has moved to 633 Park Avenue. He is taking a vacation at Point Abino, Ont.

---

Dr. Charles E. Glidden of Little Falls has accepted a position on the Staff of the Jackson Health Resort at Dansville.

---

Dr. Max Breuer of Buffalo spent July in the Adirondacks.

---

Homer A. Trotter, Ph. B., M. D., of Buffalo, has left for New York City to take a post-graduate course in the Post-Graduate Medical school and the Manhattan Ear, Nose, and Throat Hospital and Medical School.

---

Dr. Oscar Oberkircher, of Buffalo, has left for New York City where he is taking special work in general surgery.

---

Dr. Hugh J. Linn, (Univ. of Penna, Medical, 1878), formerly of Buffalo, who has been in San Francisco for some years and practicing there, has just been appointed to a professorship in the College of Physicians and Surgeons of San Francisco.

---

Two diamond stick pins and a gold ring were stolen from the residence of Dr. John H. Pryor of Buffalo about the middle of July, the exact date being unknown.

---

The residence of Dr. Charles W. Clendenan of N. Tonawanda was damaged by fire to the amount of $500 early in the morning of July 18.

---

Dr. Frank E. Brundage of Buffalo took an auto trip to Pennsylvania the latter part of July.

---

Dr. Milton H. Goldberg of Buffalo toured to Albany, N. Y., Boston and Philadelphia in July.

---

Dr. I. Harris Levy of Syracuse succeeds the late Henry L. Elsner as Prof. of Medicine in Syracuse University.

